# Episodic Migraine in the Pediatric Population: Behavioral Therapies and other Non-Pharmacological Treatment Options

**DOI:** 10.1007/s11916-025-01366-3

**Published:** 2025-03-03

**Authors:** Parisa Gazerani

**Affiliations:** 1 Department of Life Sciences and Health, Faculty of Health Sciences, Oslo Metropolitan University, Oslo, Norway; 2 Department of Health Science & Technology, Faculty of Medicine, Aalborg University, Gistrup, Denmark

**Keywords:** Migraine, Pediatrics, Adolescents, Episodic, Non-pharmacological

## Abstract

**Purpose of Review:**

The purpose of this review is to present current evidence on the multifaceted approach required for managing pediatric migraine. This includes identifying migraine triggers, managing acute pain episodes, and implementing preventive strategies. The review focuses on non-pharmacological interventions, such as behavioral and lifestyle modifications. By exploring these aspects, the review seeks to provide a comprehensive understanding of effective migraine management in children and adolescents.

**Recent Findings:**

Non-pharmacological treatments like cognitive-behavioral therapy (CBT), relaxation techniques, and biofeedback are effective alternatives to medication. Nutraceuticals and dietary considerations, including ketogenic diet, alongside education and regular follow-ups, optimize outcomes. Integrating tools for tracking migraine patterns and training for clinicians, parents, and patients enhances treatment effectiveness. Engaging adolescents and their families through education and active participation is essential for improving their quality of life.

**Summary:**

This review presents available evidence of non-pharmacological strategies for managing episodic migraine in pediatrics. CBT and lifestyle modification are documented for their effect. Future research is required to create evidence-based, comprehensive treatment plans including these and other non-pharmacological strategies tailored to individual needs.

## Introduction

Headaches rank among the top five health issues in children [1], affecting up to 58.4% of children and adolescents at some point [2]. Their prevalence has been rising globally [3], significantly impacting quality of life, social interactions, and school performance. A 2021 study [4] highlighted the importance of understanding adolescents’ perspectives on headaches to enhance interventions. Factors such as genetics, psychosocial, and behavioral aspects are often linked to pediatric headaches [3]. Despite their widespread occurrence and substantial social and economic impact, headache prevention and treatment in young people receive insufficient attention [5].

Effective headache management includes both preventive measures and treatments for acute episodes. Over-the-counter medications [6] like ibuprofen or acetaminophen are commonly used for mild to moderate headaches, including chronic migraine, while triptans [7] may be prescribed for severe migraine. Identifying and managing triggers such as stress, sleep disturbances, certain foods, dehydration, and environmental factors can help reduce headache frequency. Non-pharmacological approaches [8] like resting in a quiet, dark room, cold compresses, and hydration can also be beneficial.

Preventive strategies include maintaining regular sleep patterns, a balanced diet, adequate hydration, and stress management techniques. Lifestyle modifications [9] are crucial for preventing headaches during developmental years, improving patients’ quality of life, and reducing headache severity into adulthood. A comprehensive approach to diagnosing and treating headaches in children and adolescents is essential, with attention to serious symptoms and the effectiveness of various treatments [10].

This review focuses on managing episodic migraine in children and adolescents [11], emphasizing a balanced approach that includes medication, lifestyle changes, and education[12]. While behavioral therapies and non-pharmacological treatments are highlighted, readers can refer to recent reviews [13–16] for updated pharmacological strategies. It is important to note that there is currently no cure for migraine, which poses challenges due to limited medication options and potential side effects.

## Migraine and Episodic Migraine

Headaches are a common issue among youth, categorized into primary (e.g., migraine) and secondary types related to conditions like head trauma [5]. Migraine is the most frequent acute and recurrent headache in children, affecting 2–5% of preschoolers, 10% of school-aged children, and 20–30% of adolescent girls [11]. Often, migraine runs in families and significantly impact children’s quality of life. Unlike adults, children’s migraine is typically shorter (minimum 2 h) and often bilateral before adolescence. Symptoms vary by age: infants with colic may have a higher migraine risk, preschoolers may appear ill and pale with abdominal pain, vomiting, and a need to sleep, while children aged 5–10 often have bilateral frontal headaches with nausea, abdominal pain, and sensitivity to light and sound. Middle schoolers may experience auras, and older adolescents often have bitemporal or unilateral headaches with variable pain locations and intensities. Auras, affecting 10–20% of children, usually begin after age 8, preceding headaches by less than 60 min and lasting 5–20 min. Visual disturbances are the most common auras, including blurred vision and zigzag lines. Migraine in children is associated with psychiatric symptoms like depression and anxiety, epilepsy, motion sickness, intermittent vertigo, and cardiovascular reactivity, leading to dizziness or orthostatic intolerance. Sleep disturbances, a common trigger, are frequent in children with migraine, who may also suffer from sleep-disordered breathing and parasomnias [1, 11, 17–20].

Episodic migraine in children usually lasts 2 to 72 h but are often under 4 h, sometimes as short as 10–20 min. These headaches are milder than those in adults, with pulsating pain. Symptoms include cold extremities, nausea, vomiting, dizziness, chills, sweating, ataxia, numbness, light and sound sensitivity, memory loss, and confusion. Children often struggle to concentrate during and after migraine attacks, finding relief in sleep, followed by a postdromal phase of exhaustion or lethargy [11, 21]. Episodic syndromes, potential migraine precursors, often evolve into more typical migraine as children age [21]. Cyclic vomiting syndrome, with a prevalence of 1.9% in white populations, starts in toddlers and typically resolves by adolescence. Episodes occur every 1 to 3 months, often at night or early morning, lasting 1 to 10 days. Symptoms include abdominal pain, nausea, retching, anorexia, pallor, lethargy, light and sound sensitivity, and headaches. Abdominal migraine, characterized by recurrent abdominal pain with nausea and vomiting, often without a headache, affects 2–4% of children and may alternate with or develop into typical migraine as they grow [22, 23].

## Evaluation and Diagnosis of Pediatric Migraine

Children with migraine typically have at least five attacks featuring moderate to severe headaches, sensitivity to light, noise, and odors, nausea, and relief from rest. Their neurological exams should be normal. Neuroimaging is usually unnecessary for children with a migraine-consistent history and normal exams but should be considered for those with seizures, recent head trauma, significant headache pattern changes, focal neurological deficits, or papilledema. There are no absolute rules for neuroimaging; it relies on clinical judgment. Electroencephalography is not routinely useful but may be considered for atypical auras, episodic loss of consciousness, or seizure-like symptoms. Lumbar puncture is warranted if meningitis, encephalitis, subarachnoid hemorrhage, or pressure-related headache syndromes are suspected [11, 24].

Pediatric migraine can evolve into resistant or refractory forms, posing management challenges [25]. Resistant migraine does not respond well to conventional medications but can be managed with specific pharmacological treatments, requiring specialized care and tailored regimens. Refractory migraine does not respond satisfactorily to any treatment, necessitating a comprehensive approach addressing physiological, behavioral, and psychological factors. This multidimensional approach includes both migraine-specific medications and non-pharmacological treatments [25, 26].

Early and effective management is crucial to prevent resistant and refractory migraine. Proactive and comprehensive treatment from the onset can reduce the risk of progression. Research focuses on developing predictive tools to identify patients at risk of refractory migraine, allowing for personalized treatment plans and potentially altering the disease course. Standardized protocols for these treatments will facilitate their wider acceptance and implementation in clinical practice [13, 27].

## Treatment of Pediatric Migraine

While the biomedical approach dominates migraine treatment, a biopsychosocial model considers the individual holistically, potentially enhancing therapeutic outcomes [28]. This approach recognizes the interplay between biological, psychological, and social factors in migraine management, advocating for tailored treatments that integrate non-pharmacological strategies with conventional medical approaches. By addressing the multifaceted nature of migraine, this model aims to optimize patient care and improve treatment outcomes [28, 29].

### General Considerations

Effective migraine treatment for children involves identifying triggers, managing pain during headaches, and using preventive medications. For mild and infrequent migraine, treatment includes rest, avoiding triggers, and using analgesics as needed. It is crucial to explain migraine to the child and parents and rule out secondary causes. Ensuring adequate sleep, regular meals, proper hydration, and avoiding an overloaded schedule is essential. Identifying triggers can help reduce migraine frequency but may not eliminate headaches. Psychological triggers include stress, anxiety, and depression, while physiological triggers encompass fever, illness, missed meals, fatigue, and sleep deprivation. Environmental triggers include bright or flickering lights, barometric pressure changes, strong odors, computer screens, temperature changes, and visual patterns like stripes. Physical overexertion, minor head trauma, and travel can also trigger migraine. It’s important to note that while stress can exacerbate migraine, migraine is not a psychological illness [30].

During a migraine, children should rest in a cool, dark, and quiet room, ideally sleeping, as sleep is a potent treatment. Applying ice or gentle pressure to the painful area can also provide relief [31]. For mild to moderate attacks, nonsteroidal anti-inflammatory drugs (NSAIDs), such as ibuprofen (up to 10 mg/kg), are effective when taken early [31]. Occasionally, carbonated beverages and caffeine can enhance the absorption of some medications, including paracetamol, although this effect is inconsistent [32]. However, the use of caffeine and caffeinated beverages is not recommended for children under the age of 12 [33]. Promethazine may be used to prevent and control symptoms like motion sickness, nausea, vomiting, and dizziness. However, studies on the efficacy and safety of promethazine in pediatric populations are limited, particularly for children under two years of age, where safety and efficacy remain unclear [34]. Rectal promethazine can help with nausea and vomiting but must be administered with caution, ensuring safe and effective use in this population [31, 35]. For moderate to severe attacks, over-the-counter analgesics or triptans may be necessary [11, 31]. In emergencies involving severe or prolonged migraine, parenteral medications such as intravenous fluids, prochlorperazine, or ketorolac may be required [36, 37]. While pharmacological options may be essential in some cases, a comprehensive discussion of these treatments [11, 14, 38] is beyond the scope of this article, which focuses on behavioral and non-pharmacological approaches to managing episodic migraine in pediatric populations.

Preventive medications aim to reduce migraine frequency and severity, particularly for chronic migraine. These medications are recommended for children with frequent, prolonged, or disabling migraine unresponsive to other treatments. Common preventive drugs include amitriptyline, propranolol, gabapentin, topiramate, flunarizine, verapamil, and riboflavin. Weight gain is a concern with tricyclic antidepressants like amitriptyline, especially in teenagers. The Childhood and Adolescent Migraine Prevention (CHAMP) study [39] found that placebo responses were nearly 60%, and both topiramate and amitriptyline did not outperform the placebo. The best evidence supports using topiramate or amitriptyline combined with cognitive behavioral therapy for preventing migraine in children [38].

Non-pharmacological treatments, such as self-relaxation techniques, biofeedback, and self-hypnosis, can be effective alternatives, particularly in adolescents [12, 40, 41]. Addressing mood problems and anxiety is crucial, as these issues can hinder headache control. Environmental factors, like changes during the academic year, can impact headache burden. Many patients experience symptom improvement during summer but worsening headaches at the start of the school year. School absenteeism and impaired life functioning are significant challenges. A holistic approach to managing pediatric migraine is optimal for satisfactory outcomes [42].

### Non-pharmacological Treatments

A 40-year longitudinal study of pediatric migraine patients [43] found that migraine typically begins around age 6. During puberty or young adulthood, 62% of children had periods of being migraine-free for at least two years, but 33% experienced a recurrence after an average of six migraine-free years. Surprisingly, 60% still had migraine after 30 years, and 23% never had a migraine-free year. Among those who became parents, 52% had children with recurrent migraine. These findings highlight the need for non-pharmacological treatment strategies alongside pharmacological options [43].

A comprehensive review [44] examined non-pharmacological interventions for managing headaches in young individuals, analyzing 11 controlled studies from January 2010 to July 2018, involving 613 patients aged 10.2 to 15.7 years. The studies showed significant reductions in headache frequency (34–78%) and improvements in secondary outcomes such as disability, quality of life, depression, and anxiety. The review underscores the effectiveness of non-pharmacological treatments and emphasizes the need for further research to enhance evidence quality, including randomized patient allocation, blinded outcome evaluation, consistent use of headache frequency as the primary endpoint, longer follow-up durations, exploration of biomarker changes, and predictive models [44].

Migraine management should be individualized, considering medication and other factors influencing the disease. The biopsychosocial (BPS) model [28] views migraine as multifaceted, addressing biological, psychological, and social aspects. Treatment should focus on pain relief and preventive measures based on migraine frequency and impact on daily life. A holistic approach involves evaluating all aspects of an individual’s life, including biological, social, psychological, and environmental influences. Improving quality of life, rather than just reducing pain, should be a priority, with patient-centered communication to address stigma and enhance management [28, 29].

Individuals with migraine often struggle with accepting and coping with pain, leading to high levels of perceived disability and pain catastrophizing. The unpredictable nature of severe migraine attacks can create a sense of loss of control over the illness [45]. Coping strategies [46] such as monitoring migraine patterns and triggers, managing painkiller use, and education on lifestyle factors (weight management, stress reduction, regular sleep patterns, and physical exercise) are crucial.

Non-pharmacological approaches to managing migraine [47] include various methods such as non-invasive neurostimulation techniques, behavioral therapies like CBT and mindfulness-based stress reduction (MBSR), nutraceuticals, and complementary and alternative medicine (CAM) modalities like traditional Chinese medicine and massage. It is advisable to consult healthcare providers before starting these treatments to ensure safety and avoid potential interactions with other therapies. Behavioral therapies targeting stress management, medication overuse, reducing catastrophizing, and enhancing self-efficacy can effectively complement pharmacological treatments. Social factors, though difficult to change, play a significant role in migraine patterns. Assessing cultural, environmental, and socioeconomic factors, and fostering a positive relationship between migraine sufferers and healthcare providers, can improve care effectiveness. Joining support groups can help individuals with migraine connect with others facing similar challenges and gain external support. Non-pharmacological treatments can offer significant benefits, including minimal risk of adverse effects compared to pharmacological options, which are known for side effects like sedation and behavioral changes. These treatments can empower young patients to develop coping skills and reduce reliance on medications. Further research is needed to understand the mechanisms underlying these treatments’ efficacy and to address methodological limitations. Randomized controlled trials with longer follow-up periods and investigation into predictive factors are crucial to strengthen the evidence base for non-pharmacological interventions in pediatric headache care.

#### Neurostimulation

Neuromodulation [48–50], altering the nervous system via electric, magnetic, or chemical stimulation, has ancient roots with electric eels, and modern devices like transcutaneous supraorbital neurostimulator (tSNS) and the single-pulse transcranial magnetic stimulator (sTMS) are food and drug administration (FDA)-approved but mainly for adult migraine. Single-Pulse Transcranial Magnetic Stimulation (sTMS) shows promise in reducing migraine frequency and severity in adolescents. The eNeura Transcranial Magnetic Stimulator, delivering a single magnetic pulse, is FDA-approved for migraine with aura and recently for children. In 2019, eNeura received FDA approval for adolescent acute and preventive migraine treatment [51]. Although safety is established in children, efficacy remains unproven. Adolescent studies on sTMS showed good tolerance but lacked efficacy assessment, prompting ongoing research for pediatric efficacy. Non-invasive vagus nerve stimulation (nVNS) targets the vagus nerve, useful for children intolerant to pharmacological treatments. Electrical Trigeminal Nerve Stimulation (eTNS) stimulates the trigeminal nerve, reducing migraine frequency, well-tolerated in children. Remote electrical neuromodulation (REN), exemplified by Nerivio®, is an FDA-approved wearable device for acute/preventive migraine treatment in patients aged 12 + . Adolescent REN [52] usage for acute attacks correlates with reduced monthly treatment days, suggesting preventive benefits for migraine in this age group.

#### Psychological Interventions

Recent reviews have underscored the importance of psychological interventions in managing pediatric headache disorders, which significantly impact children’s well-being and quality of life. CBT, biofeedback, and mindfulness-based therapies have demonstrated efficacy in reducing headache frequency and improving the quality of life for children and adolescents [53]. These interventions are tailored to individual developmental stages and have shown promising results in various clinical settings.

CBT teaches coping skills, stress management, and relaxation techniques tailored to children’s developmental stages. Combination therapy with analgesics has shown reduced medication needs [54, 55]. When added to standard antimigraine therapy, CBT has also shown remarkable efficacy in pediatric migraine therapy, demonstrating one of the strongest levels of evidence among treatment modalities. Related to the beneficial effects of CBT, most available studies are for chronic migraine [38]. Powers et al. [56] conducted a randomized controlled trial involving children aged 10–17 with chronic migraine, showing that those who received CBT along with preventive medication (amitriptyline) experienced improved PedMIDAS scores and fewer headache days compared to those treated with medication alone. In another study, Blume et al. [57] conducted a retrospective analysis involving children aged 8–18 with chronic migraine who received two or more biofeedback sessions. The analysis revealed a statistically significant reduction in headache days among participants. Furthermore, there are numerous free relaxation apps available for smart devices, offering accessible resources for practicing relaxation techniques. A review [58] has looked into the apps for calming, relaxation, and mindfulness for pediatric palliative care. These can potentially be adjusted for use in migraine care in the pediatric population. Mindfulness techniques, such as mindfulness-based stress reduction (MBSR) and meditation, enhance pain coping and stress management. Biofeedback empowers children to control physiological processes like muscle tension, reducing headache frequency.

Bio-behavioral approaches, including biofeedback and multidisciplinary behavioral programs, effectively manage headache pain [53]. Multidisciplinary programs involving experts in physical medicine, psychology, and neurology enhance functioning and pain control. Recognizing and monitoring mood changes in children with migraine is crucial for effective management. Interventions combining pharmacological and non-pharmacological approaches are recommended to reduce disability and prevent chronification.

Acceptance and commitment therapy (ACT), a mindfulness-based therapy emphasizing acceptance and psychological flexibility, shows promise in migraine treatment [59]. Unlike traditional therapies, ACT focuses on developing a different relationship with difficult thoughts and feelings, fostering self-acceptance and non-judgmental observation of sensations. It can be delivered individually or in groups, making it adaptable for various clinical populations.

Virtual group sessions, especially amid the COrona VIrus Disease of 2019 (COVID-19) pandemic, offer immediate validation and support for individuals with shared pain conditions. Delivering ACT through telehealth platforms enhances accessibility and minimizes migraine triggers. Future research aims to assess patient satisfaction, coping strategies, and outcomes related to migraine disability and the hypothalamic-pituitary-adrenal axis (HPA-axis) function, with data informing large-scale trials and future treatment practices.

### Acupuncture

Acupuncture emerges as a promising and safe treatment for pediatric migraine. A 2023 review [2] revealed that although few guidelines explicitly recommend acupuncture for children, the available evidence supports its efficacy. The review, drawing from eight studies selected from 135 papers, indicated that acupuncture positively impacts both headache frequency and intensity while being well-tolerated. Notably, while the long-term efficacy of acupuncture remains unexplored, consistent positive effects on headache frequency and intensity were observed across variations in tools, procedures, and application sites.

However, to better evaluate acupuncture’s efficacy, tolerability, and long-term outcomes in children and adolescents, additional studies with larger sample sizes and standardized procedures are imperative. Such research endeavors are critical for developing guidelines and ensuring the availability of safe, effective treatment options for pediatric patients grappling with recurrent and debilitating headaches.

### Nutraceuticals

Based on a recent narrative review [12], non-pharmacological approaches in pediatric migraine treatment are gaining attention. Grazzi et al. [60] provide an overview of nutraceutical options like Feverfew and Riboflavin, which are considered valuable, especially for patients with medication overuse or contraindications to drug treatment. However, further research is needed to assess their long-term effectiveness. Vitamin B2 [61], magnesium [62], and melatonin have shown efficacy in preventing migraine attacks. Melatonin [63] has even demonstrated superiority over placebo and amitriptyline in adult trials, with ongoing trials investigating its potential in adolescents. Riboflavin has also shown effectiveness in randomized controlled trials, while magnesium supplementation has been found to reduce headache frequency in children.

A review of clinical studies from 2010 to 2019 [64] explored prophylactic therapy for pediatric migraine, including non-pharmacological approaches, nutraceuticals, and herbals. Limited evidence exists regarding the efficacy of nutraceuticals in pediatric migraine prophylaxis, with few randomized controlled trials (RCTs) available. Previous RCTs on substances like magnesium, riboflavin, feverfew, and hydroxytryptophan did not yield conclusive results. However, a recent RCT investigating coenzyme Q10 (CoQ10) showed a significant reduction in migraine frequency, severity, and duration, though similar effects were observed in both the placebo and CoQ10 groups [65].

Ginkolide B, an antagonist of platelet-activating factor (PAF) receptors, has been studied in combination with other nutraceuticals in pediatric open-label studies [66]. These studies demonstrated a significant reduction in the number of migraine attacks after treatment with a combination of ginkgolide B, CoQ10, riboflavin, and magnesium. Another open-label study compared the efficacy of this combination with a complex including L-tryptophan, 5-hydroxytryptophan, and vitamins, showing significant reductions in headache attack frequency for both combinations, with a more pronounced effect observed with the ginkolide B-containing complex [67, 68].

### Dietary and Lifestyle Considerations

Pediatric migraine poses unique challenges due to occurring during incomplete brain development, necessitating tailored treatment approaches [69–71]. Non-pharmacological strategies, particularly lifestyle modifications, are integral for effective management. Lifestyle changes, such as establishing healthy sleep patterns, maintaining hydration, regular exercise, and minimizing light stimulation, are vital in migraine prevention. Notably, dietary habits are influential, with recent research highlighting the gut-brain connection [72]. Dietary therapies, including the ketogenic diet and its variations, show promise in pediatric migraine management by addressing dysbiosis, reducing inflammation, and enhancing mitochondrial function [72]. While studies on diet therapy in pediatric migraine are limited, insights from adult research are valuable. For instance, studies on ketogenic diets (KD) demonstrate significant reductions in migraine days, headache intensity, and medication intake [73]. Research on KD and its variants as therapies for neurological diseases has grown rapidly since 1995. A systematic review by Caminha et al. in 2022 [73] demonstrates that KD and its variants are effective migraine prevention therapies in both adolescents and adults.

Additionally, low glycemic index diets have shown efficacy in migraine management, with digital methods aiding individualized patient care [74]. Lelleck et al. explored the individual management of a low glycemic index diet (LGIT) in patients with migraine, (including teenagers of 18 and 19 years old) using digital methods and showed that sinCephalea as a non-pharmacological digital migraine prophylaxis can be useful to deliver a therapeutic effect within the range of pharmacological interventions [74]. Pasca et al. [75] reported successful sleep stabilization in a pediatric migraine patient through KD, confirmed by polysomnography.

The incomplete development of the brain and gastrointestinal tract in adolescents underscores the potential effectiveness of nutritional interventions like diet therapy in managing pediatric migraine. A neuro-nutritional team comprising pediatric neurologists, nurses, and nutritionists is crucial for developing and managing tolerable diets tailored to individual patient needs [72]. Collaborative efforts among these professionals facilitate the implementation of dietary therapies, optimizing outcomes for pediatric migraine patients.

Diet is not the only component. Lifestyle modifications are fundamental in managing primary headaches like migraine [9]. Good sleep hygiene, including a consistent bedtime routine and limiting screen time before bed, is crucial for migraine management. Regular sports in a non-competitive group setting, relaxation techniques, and avoiding high goal pressures are recommended for adolescents with migraine [11]. Adequate hydration, regular meals, and good sleep hygiene are crucial, with the American Academy of Pediatrics suggesting 60–80 oz of fluids daily for children aged 8 years and above [14].

Beyond the physical symptoms, migraine sufferers endure non-neurological burdens, including stigma, social isolation, and anxiety. Anxiety, more prevalent than depression, often manifests as anticipatory anxiety about future attacks and can exacerbate migraine symptoms. This bidirectional relationship suggests that anxiety may contribute to migraine progression, for example from episodic to chronic. Therefore, complementary and integrative health approaches, such as mind–body therapies, which have shown effectiveness in managing pain and improving quality of life can also be considered. In this line, educating parents, the pediatric population affected by migraine, and their school or sports instructors is important.

There is growing recognition that the burden of migraine extends beyond the headache phase of the attack, often persisting between episodes in what is referred to as the interictal phase [76], during which patients may experience symptoms such as allodynia, hypersensitivity, photophobia, phonophobia, osmophobia, visual or vestibular disturbances, and motion sickness. These subtle interictal clinical manifestations, along with the anxiety of unpredictable migraine attacks, can significantly impact a patient’s quality of life [76]. Despite the importance of this phase, there are currently few tools available to assess the interictal burden that can influence an optimal and comprehensive treatment [76].

### Digital Medicine

mHealth studies are at an advantage over some other behavioral studies in being able to monitor usage patterns by tracking the frequency, duration, and length of the intervention. In addition, mHealth interventions have high fidelity because the same intervention is offered to all the participants. A study in adults [77] demonstrated that in 51 patients, more than half of whom had severe migraine, about one of every two patients demonstrated engagement with smartphone-based progressive muscle relaxation (PMR) intervention based upon a brief, initial introduction to the app. App use was associated with a reduction in headache days. Higher depression scores were negatively correlated with diary and PMR use, while higher anxiety scores were positively correlated [77]. However, there appeared to be a time-limited acceptability of the intervention by 6 weeks. This is a promising area given its low cost, scalable method, and future studies can begin to examine efficacy. Self-management of migraine in adolescents is complex and significantly impacts health outcomes. Using an iterative co-design process, the Migraine manager digital self-management tool [78] was developed and optimized. An 8-week single-arm open-label trial enrolled adolescents aged 11–18 years and analyzed data. The primary outcome measured was headache days. The Migraine Manager tool demonstrated feasibility and preliminary efficacy, suggesting it could benefit adolescents with migraine who might not otherwise receive such services. Larger controlled trials with long-term follow-up are needed to confirm its clinical efficacy. Digital therapeutics (DTx) in general can offer promising solutions for unmet behavioral health needs. During the Covid-19 pandemic, digital therapeutics were considered as treatment option for migraine management[79]. Emerging clinical research on DTx shows an upward trend, with many studies highlighting its feasibility and effectiveness in tracking and recording migraine attacks, aiding self-diagnosis, and treatment, and providing long-term management guidance. However, larger studies are needed to confirm the effectiveness of DTx in migraine management [79].

### Art Therapy

Art therapy shows promising potential in the treatment of migraine [80]. Its efficacy in managing other types of pain suggests it could be beneficial for migraine [81]. This non-pharmacological approach is safe, cost-effective, and offers the benefits of group therapies while potentially carrying less stigma. Avoiding terminology associated with mental healthcare might improve patient participation. Group art therapy can also provide a sense of community, reducing the isolation many migraine sufferers feel. Art therapy’s visual aspect allows patients to express their pain, making it visible to themselves, their families, and providers. This expression can enhance self-efficacy and help patients develop long-term coping skills for managing migraine [80]. A recent study in adults [82] assessed the impact of the Mindfulness-Based Art Therapy Program on participants’ depression, anxiety, stress symptoms, and happiness. Data analysis showed that the program effectively reduced depression and stress symptoms and increased happiness in participants, although it did not significantly impact anxiety symptoms. While these initial findings are promising, further research is needed to validate the effectiveness of the Mindfulness-Based Art Therapy Program as an alternative treatment for migraine patients [82] in various age groups. Various forms of art remain to be explored for their efficacy in treating episodic pediatric migraine.

### Education

A recent review [30] has looked into the triggers of pediatric migraine with the aim of improving headache education. Migraine significantly affects the quality of life in children and adolescents, impacting not only the individuals but also their families. In assessing the current evidence on factors correlated with migraine in children and adolescents, three primary triggers or risk factors were identified: stress, sleep poverty, and alimentation (including diet and obesity). Stress management techniques, including relaxation exercises, mindfulness, and CBT, are recommended to help reduce migraine incidence in children and adolescents. Evidence suggests that improving sleep hygiene, ensuring regular sleep schedules, creating a restful sleeping environment, and minimizing screen time before bed can significantly reduce migraine episodes. Clinicians should encourage a balanced diet, regular meals, and weight management rather than restricting specific foods unless a clear dietary trigger is identified.

Table 1 summarizes the non-pharmacological therapeutic approaches for pediatric migraine.

**Table 1:** Non-pharmacological therapeutic approaches for pediatric migraine

Lifestyle and Nutraceuticals	Non-Invasive Neuromodulation	Mind–Body Therapies	Miscellaneous
Diet and Lifestyle - Food Triggers - Obesity/Overweight - Exercise and Activity - Ketogenic Diet - Digital/screen time	- eTNS (Transcutaneous supraorbital electrostimulation and external trigeminal nerve stimulator) - nVNS (Non-invasive vagal nerve stimulator) - TMS (Transcranial magnetic stimulator) - REN (Remote electrical neuromodulation)	- Biofeedback treatment - CBT - Mindfulness	- Acupuncture - Education - Digital Medicine - Art Therapy
Sleep - Sleep hygiene			
Supplements - Magnesium - Riboflavin - Melatonin - Vitamin D			

## Conclusion

Episodic migraine in children, when effectively managed, can lead to significant improvements in quality of life and daily functioning. Effective treatment involves identifying potential triggers, managing pain during headaches, and using preventive strategies. For children with mild, infrequent migraine, treatment primarily includes rest, avoiding triggers, and using analgesics as needed. Key strategies include education, lifestyle adjustments (e.g., adequate sleep, regular meals, proper hydration), avoiding overloading the child’s schedule, helping the child identify potential migraine triggers, and setting realistic expectations. Recognizing the role of psychological factors such as stress, anxiety, and depression is also important. Environmental triggers such as fluorescent lights, bright or flickering lights, barometric pressure changes, high altitude, strong odors, computer screens, and rapid temperature changes must also be recognized and minimized or avoided. The goal of these strategies is to manage and reduce the frequency of migraine headaches in children, providing them with a better quality of life.

Non-pharmacological treatment options, including CBT, relaxation techniques, and biofeedback, are effective interventions for preventing migraine in adolescents and can serve as viable alternatives to medication [83]. Nutraceuticals, non-invasive neuromodulation, and behavioral therapies, in addition to education, self-management strategies, and regular follow-ups, can optimize migraine treatment outcomes toward holistic care for migraine patients [12]. Care providers must understand complementary and integrative treatments, their efficacy, benefits, and risks to discuss them with young patients and their families. Such conversations can empower patients, strengthen therapeutic relationships, and improve outcomes and patient-centered care [84]. Identifying which patients benefit most from specific non-pharmacological treatments remains essential, supporting the goals of precision medicine [85].

Given the limited and inconclusive evidence, establishing a comprehensive headache education plan focused solely on behavioral and lifestyle interventions is challenging. However, clinicians can still provide valuable guidance. For example, they can encourage stress management through techniques such as mindfulness, relaxation techniques, and counseling. They can also advise on sleep hygiene by encouraging the maintenance of consistent sleep schedules, optimizing sleep environments, and limiting screen exposure before bedtime. Maintaining a balanced diet, avoiding skipping meals, and focusing on healthy eating patterns should be encouraged in pediatric migraine management, with specific food restrictions considered only if clear triggers are identified.

The development of a standardized educational and cognitive treatment manual is crucial for advancing pediatric migraine management. By focusing on evidence-based interventions and a personalized approach, future research can help create effective, comprehensive treatment plans that improve the quality of life for affected children and adolescents. Although current guidelines offer some direction on behavioral and lifestyle interventions, the evidence supporting these recommendations is not robust. Therefore, future larger, prospective studies are needed to design a standardized educational and cognitive treatment manual that will contribute to the development of more effective pediatric migraine treatments. Investigating the long-term outcomes of these interventions to ensure sustainable benefits is another crucial goal.

A comprehensive educational and cognitive treatment manual should be based on robust evidence and include standardized protocols for stress management, sleep hygiene, dietary guidance, and other relevant lifestyle factors. Integrating the BPS model to tailor treatments to the individual needs of pediatric patients is highly important. Clear, age-appropriate information about migraine, potential triggers, and management strategies should be provided, with resources available to parents and caregivers to support their children’s migraine management. Adolescents can be educated through interactive tools and digital platforms that are engaging and rewarding. These platforms can also be equipped with online evidence-based educational materials for stress management techniques such as mindfulness, relaxation exercises, and CBT, as well as guidance on maintaining proper diet, exercise, sleep hygiene, and managing screen time.

Integrating tools for tracking migraine patterns, triggers, and treatment effectiveness, such as electronic headache diaries, will be useful. Establishing protocols for regular follow-ups and adjustments to treatment plans based on patient progress would be beneficial. Training should also extend to clinicians and parents or caregivers to effectively deliver the standardized treatment manual and support patients. Engaging both patients (mainly adolescents) and their families in the treatment process through education and active participation in managing migraine is essential.

## Data Availability

No datasets were generated or analysed during the current study.
